# Exercise and asthma: an overview

**DOI:** 10.3402/ecrj.v2.27984

**Published:** 2015-11-03

**Authors:** Stefano R. Del Giacco, Davide Firinu, Leif Bjermer, Kai-Håkon Carlsen

**Affiliations:** 1 Department of Medical Sciences “M. Aresu”, University of Cagliari, Cagliari, Italy; 2 Department of Respiratory Medicine and Allergology, Lund University, Lund, Sweden; 3 Oslo University Hospital, Oslo, Norway

**Keywords:** exercise-induced asthma, exercise-induced bronchoconstriction, EIA, EIB, sports, allergy

## Abstract

The terms ‘exercise-induced asthma’ (EIA) and ‘exercise-induced bronchoconstriction’ (EIB) are often used interchangeably to describe symptoms of asthma such as cough, wheeze, or dyspnoea provoked by vigorous physical activity. In this review, we refer to EIB as the bronchoconstrictive response and to EIA when bronchoconstriction is associated with asthma symptoms. EIB is a common occurrence for most of the asthmatic patients, but it also affects more than 10% of otherwise healthy individuals as shown by epidemiological studies. EIA and EIB have a high prevalence also in elite athletes, especially within endurance type of sports, and an athlete's asthma phenotype has been described. However, the occurrence in elite athletes shows that EIA/EIB, if correctly managed, may not impair physical activity and top sports performance. The pathogenic mechanisms of EIA/EIB classically involve both osmolar and vascular changes in the airways in addition to cooling of the airways with parasympathetic stimulation. Airways inflammation plays a fundamental role in EIA/EIB. Diagnosis and pharmacological management must be carefully performed, with particular consideration of current anti-doping regulations, when caring for athletes. Based on the demonstration that the inhaled asthma drugs do not improve performance in healthy athletes, the doping regulations are presently much less strict than previously. Some sports are at a higher asthma risk than others, probably due to a high environmental exposure while performing the sport, with swimming and chlorine exposure during swimming as one example. It is considered very important for the asthmatic child and adolescent to master EIA/EIB to be able to participate in physical activity on an equal level with their peers, and a precise early diagnosis with optimal treatment follow-up is vital in this aspect. In addition, surprising recent preliminary evidences offer new perspectives for moderate exercise as a potential therapeutic tool for asthmatics.

Exercise-induced respiratory symptoms were first described by Araeteus the Cappadocian in the 1st century A.D. (*‘if from running, gymnastics, or any other work, breathing becomes difficult, it is called “Asthma”’*). In the ‘modern’ era, Jones et al. (1) firstly described in 1962 the effects of exercise on ventilatory function in children, together with systematic exercise tests.

The terms ‘exercise-induced asthma’ (EIA) and ‘exercise-induced bronchoconstriction’ (EIB) are often used interchangeably. A consensus between the American Academy of Allergy, Asthma and Immunology (AAAAI), the American College of Allergy, Asthma and Immunology (ACAAI), and the Joint Council of Allergy, Asthma and Immunology (JCAAI) used the term ‘EIB with asthma’ for exercise-induced bronchoconstriction (EIB) with clinical symptoms of asthma and ‘EIB without asthma’ for an acute airflow obstruction without asthma symptoms (2). A joint Task force of European Academy of Allergy and Clinical Immunology and European Respiratory Society defined EIA as symptoms of asthma occurring after heavy exercise, whereas EIB denoted the reduction in lung function occurring after exercise, as seen in a standardized exercise test (3). In this review, we will refer to EIB as the bronchoconstrictive response while EIA also includes EIB + asthma symptoms. The two conditions will be discussed separately when the populations are specifically identified; otherwise, they are collectively referred to as EIB/EIA.

Aim of this review is to provide the reader with the current knowledge on this topic, focusing in particular on the athlete's asthma phenotype, together with a paragraph on new perspectives for exercise as a therapeutic tool for asthmatics.

It has been claimed that up to 75–80% of asthmatic subjects without anti-inflammatory treatment may experience an asthma attack provoked by exercise (4), but also individuals without a diagnosis of asthma may experience a significant reduction in lung function after heavy exercise, sometimes representing a risk factor for the development of asthma (5). Whereas the physiologic response to exercise usually result in slight bronchodilation, in population-based studies individuals without an asthma diagnosis may also suffer from EIB (6).

A minimum of 5–8 min continuous high-intensity effort is required to develop an exercise-induced bronchoconstrictive response. EIB is usually observed 2–10 min after heavy exercise, and not during maximum exercise intensity. However, in a study from Van Leeuwen et al., EIB occurred in children during sub-maximal exercise, not after (7).

Children and adolescents are more frequently affected than adults (8) and in the Oslo birth cohort study ‘Environment Childhood Study’ 36.7% of 10-year-old children with a diagnosis of asthma showed EIB, with a positive exercise test, while 8.6% had a positive EIB test in the entire population-based birth cohort (6).

Elite athletes also have an increased risk for EIA/EIB, especially those that participate in endurance sports such as swimming, running, and cycling and in winter sports (9).

Despite this increased prevalence, it is reassuring that many asthmatic elite athletes with optimal asthma treatment are able to participate on an equal level with their peers in the Olympic Games and in other top level international competitions. Fitch even described that asthmatic athletes even succeeded to win more medals than other athletes (10).

On the other hand, it is well known that asthmatic children suffering from EIA will become passive and participate at a low level in physical activity and play. Strunk reported that physical fitness was related to self-perception and psychological functioning in asthmatic children (11). However, a more recent study showed that although mild-to-moderate asthma had a moderate impact upon daily life quality in children, it did not seem to influence psychological functioning of the child or the family (12). Thus, optimal treatment of EIA becomes as important in the child with asthma as in the elite athlete with asthma, and in the common international asthma treatment guidelines, mastering EIA during childhood is one of the main aims of treating asthmatic children (13, 14).

## EIA and EIB: pathophysiological background

Pathogenic mechanisms of EIA/EIB probably differ in the athlete compared to children, adolescent, or adult with asthma (15).

Exercise is a quantifiable and reproducible stressor that can be modified experimentally and can be considered as a model of stress (16). It has an effect on the endocrine activity and the nervous and the immune systems, thereby activating several complex interacting mechanisms within the psycho-neuro-immune-endocrine pathways (17).

Classical mechanisms behind EIA and EIB include the so-called *osmolar* (or airway drying) and *vascular* (or ‘thermal’) hypothesis. Both hypotheses are based on the marked increased ventilation during physical activity, leading to increased water and heat loss through respiration. Increased water loss increases the osmolality of the extracellular fluid lining the bronchial mucosa, causing water to move extracellularly possible through the water channels, aquaporins, and bronchial epithelial cells to ‘shrink’, with an increase of intracellular ion concentration (18) and release of inflammatory mediators from mast cells, eosinophils, neutrophils, and other inflammatory cells including newly formed eicosanoids (19, 20). The epithelium may serve as a key regulator of the balance of eicosanoids in the airways by activating the release of bronchoconstrictive eicosanoids in inflammatory cells in close contact and by alterations that reduce the synthesis of the protective PGE2 (21).

The ‘*Vascular’* or ‘*Thermal*’ hypothesis involves airway rewarming after cooling of the airways as the initiating mechanism. During normal tidal breathing, the nose functions like a rebreathing organ with warming up (up to 37°C) and humidifying the inspired air. The respiratory heat loss increases with increasing exercise intensity due to the increased ventilation. If the inhaled air is cold, the respiratory heat loss with the resulting cooling of the airways is further enhanced (15, 22, 23). The cooling of the airways results in reflex parasympathetic nerve stimulation causing bronchoconstriction through the vagal nerve (24). At first, it is notable that a reflex vasoconstriction of bronchial venules to conserve heat occurs, but when exercise ceases, the increased ventilation ceases, as does the cooling stimulus, causing a rebound vasodilatation of the peribronchial venules. The resulting smooth muscle constriction due to nerve stimulation and mucosal oedema due to vasodilatation in susceptible individuals reduces the size of the bronchial lumen with increased airways resistance (25). The main factor is now thought to be the inflammation induced by changes in airway osmolarity, and both mechanisms (osmolar and thermal) may work together under conditions of significant heat loss (3).

Also, exercise, with its repeated hyperventilation challenges, may cause bronchial epithelial damage with eosinophil and neutrophil influx and increased peptidoleukotriene concentrations in broncho-alveolar lavage fluid. In cultured human bronchial epithelial cells, experimental exposure to a hyperosmolar medium or the cooling–rewarming process is capable of triggering an inflammatory cascade by increasing the expression of various chemokines and cytokines such as IL-8 and RANTES (Regulated on Activation, Normal T cell Expressed and Secreted). This may suggest a possible mechanism for exercise-induced leukocyte migration into the airways (26, 27). Athletes of different sports show an increased number of inflammatory cells in induced sputum, when measured at rest. Furthermore, pro-inflammatory cytokines are increased after prolonged, strenuous exercise (28). This interplay between hyperventilation, hyperosmolarity, and immune changes seems capable of causing a multi-factorial bronchial inflammatory response, involving common pathways of allergic and asthmatic inflammation.

Finally, the impact of rhinitis on asthma must also be considered (29). Epidemiological studies indicate that asthma and rhinitis frequently coexist, even in the absence of atopy (30). A total of 80–90% of asthma patients may report rhinitis symptoms and 19–38% of patients with allergic rhinitis report symptoms of asthma. Rhinitis is also very frequently found in athletes (31), with a variable prevalence depending on the criteria used for diagnosis in different studies. In a Swiss study, athletes with hay fever had significantly more frequent exercise-related airway symptoms, but received inadequate treatment (32).

## Why do elite athletes develop asthma?

The prevalence of EIA is higher among elite athletes than in the general population (33), with a reported prevalence of up to 22.8% in summer sports and even higher (up to 54.8%) in winter sports. Such variability may depend on a lack of uniformity in the study methods (e.g. self-reported questionnaires, anti-doping applications for anti-asthmatic drug use, spirometry with broncho-reversibility test, etc.). However, those undertaking endurance sports seem to be at particular risk. Studies performed in US Olympic athletes show an increasing trend of the disease with 9.7% in 1976, 16.7% in 1996 (34), and 21.9% both at Nagano Winter Games (35) and at the Sydney 2000 Games (36) being reported. Between 4.2 and 7.7% of Olympic Athletes had a confirmed diagnosis of asthma with a positive bronchodilator or a bronchoprovocation test in 2006, 2008, and 2010 Olympic Games (37). In certain groups of athletes, such as swimmers and skiers, prevalence is even higher compared to the athlete population in general.

In addition to the type of sport with focus on endurance training, environmental factors are also of importance. This includes cold air for cross-country skiers and organic chlorine products for swimmers (38). The exposure to the environmental agents is further increased for these athletes due to their heavily increased ventilation during their daily repeated training and competitions (9, 39).

In elite endurance athletes, the respiratory epithelial damage and reduced repair seem to be central in the development of inflammation in the athlete's asthma phenotype (15, 40). Epithelial damage is the final result of repeated courses of intensive training sessions and competitions at the intensity of hyperpnoea necessitated by exercise at an elite level. This mechanism is partially reversible at the end of career and is also supported by evidence that transient airway hyperresponsiveness (AHR) can be associated with periods of intensive training (41–43). In addition, when other stimuli contribute to this process by causing increased epithelial damage and inflammation, such as respiratory virus infections, AHR develops and asthma symptoms may appear for the first time in previously asymptomatic athletes, as demonstrated by increased AHR for prolonged periods of time after respiratory virus infections (39).

The level of exercise load is fundamental, and its relevance was already confirmed by Carlsen et al. in 1989, with the first findings that AHR correlated with the increase in exercise load (increase in blood lactate) in both asthmatic and healthy swimmers (44). A kind of ‘biological gradient’ exists, and can be evaluated by examining exposure time in a sport or cumulative hours of training and how this modifies the risk of airway dysfunction (41). Heir and Larsen (39) reported that airway sensitivity to methacholine in cross-country skiers was negatively correlated with changes in the volume of exercise performed. In addition, Stensrud et al. (45) observed increased AHR to methacholine in elite athletes with increasing age and training volume. Other authors (46, 47) reported a correlation between training load and sputum neutrophilia. Furthermore, in young competitive rowers, the cellularity of induced sputum obtained shortly after ‘all-out’ tests correlates directly with minute ventilation during the bout (48). Recently, the finding of an increased number of basophils in the induced sputum of athletes with and without asthma, but not in controls, may suggest the role of basophils as new possible players in the peculiar features of the athlete's asthma (49).

Data in humans have been confirmed also in exercising animals, where inflammatory changes in the airways have been found. In particular, epithelial damage has been found in exercising mice (50), sledge dogs (51), and in experimental studies (52), suggesting this to be the primary lesion in asthma and EIB.

Signs of lung epithelial stress and subsequent inflammation after exercise or bronchoprovocation tests can be found in some interesting experimental studies. Increased number of bronchial epithelial cells with apoptosis in induced sputum after repeated half-marathon races, in addition to increased serum levels of Clara Cell protein 16 (CC16) and increased supernatant interleukin 8 levels in induced sputum have been found in endurance runners (53). Inhaled air humidity and temperature can influence CC16 urinary levels that have been found increased in another study, but the levels were reduced when the study subjects inhaled warm, humid air during running (54). Eucapnic voluntary hyperpnoea (EVH) test with dry air also increased urinary CC16 levels irrespective of the athletes’ positive or negative bronchoprovocation result (55). Similar findings have been found in athletes that underwent a bronchoprovocation test with mannitol (56). The increased number of columnar epithelial cells in induced sputum of asthmatic patients with EIB, compared with asthmatic patients without EIB (57), related to degree of EIB, and associated with the supernatant sputum levels of cysteinyl leukotrienes, histamine, and increased expression of MUC5AC (20).

The link between respiratory epithelial damage and increased airways inflammation is further strengthened by the role of the ‘inflammasome’ IL-1-Th17 response in asthma (58). The extracellular water movement across cell membranes is important in the mechanism of EIA. Aquaporin (Aqp) is a channel for osmotic aqueous water transport, expressed in respiratory sub-epithelial glandular cells and alveolar type 1 cells of the lungs, and mice lacking the gene for Aqp five show methacholine-induced bronchiolar hyperresponsiveness compared with normal mice (59, 60). A relationship between methacholine bronchial responsiveness and diminished pilocarpine-induced sweat secretion, tearing rate, and salivary flow rate in healthy athletes indicating an autonomic dysfunction with involvement of the parasympathetic nervous system has also been demonstrated (59).

The increased parasympathetic activity has also been demonstrated in endurance runners by Filipe et al. through pupillometry (61), by Knopfli et al. in two studies reporting higher parasympathetic nervous activity in top cross-country skiers (62, 63) and indirectly by Deal et al. through the blocking effect of atropine inhalation on EIB induced by cold air inhalation, already in 1978 (64).

## Which sport for the asthmatic?

One dilemma that physicians caring for athletic adolescents are faced with is what kind of recommendation should be given to those suffering from asthma. Athletes who participate in endurance and winter sports as well as swimming are at higher risk for EIA/EIB. Long-duration exercise and very low air temperature easily expose these athletes to the osmolar and vascular changes in the airway, fundamental in the EIA/EIB pathophysiology. Types of training and atopy are independent risk factors for EIA/EIB: combining the two factors in a logistic regression model, atopic speed, and power athletes have a 25-fold increased risk of EIA/EIB compared to non-atopic subjects, long-distance runners a 42-fold and swimmers a 92-fold increased risk (65). In addition, for the asthmatic athlete it is also important to avoid strenuous exercise during temporarily increased exposure to ‘biological stress’. This can be increased aeroallergen load, extreme cold air environment, or strenuous exercise too close to a recent viral respiratory tract infection. With an early and precise diagnosis, insightful precaution protecting the airways from extreme biological stress and an early start of anti-inflammatory treatment, the progression of bronchial hyperresponsiveness and asthma in these athletes may usually be well controlled. Data from the recent Olympic Games show that asthmatic athletes have won more medals during recent Olympic Games than athletes without asthma (10), demonstrating beyond doubt that the asthmatic athletes may compete on an equal level with their peers.

Sports with low risk for the development of asthma and bronchial hyperresponsiveness are the ones in which the physical effort is of short duration and in which high ventilatory levels are not reached. Medium-risk sports are team sports in general, in which the alternation of aerobic and anaerobic phases, as well as the relatively brief periods of continuous high-intensity exercise (in any case usually lower than 5–8 min) result in a lower risk of bronchial hyperreactivity. High-risk sports, as already stated, are endurance and winter sports in general (Table 1).

**Table 1:** Examples of sports and their potential risk of EIA/EIB

Low-risk sports	Medium-risk sports	High-risk sports
All sports in which the athlete performs a<5–8 min effort	Team sports in general, in which the continuous effort rarely lasts more than 5–8 min	All sports in which the athlete performs a >5–8 min effort and/or in a dry/cold air environment
Track and field: Sprint (100, 200, and 400 m) Middle distance (800 and 1,500 m) Hurdles (100, 110, and 400 m) Jumps Throws Decathlon Heptathlon Tennis Fencing Gymnastics Downhill skiing Boxing Golf Body building Weightlifting Martial arts	Soccer Rugby American football Basketball Volleyball Handball Baseball Cricket Field hockey	Track and field: Long distance (5,000 and 10,000 m) 3,000 m steeplechase Pentathlon (mixed) Walks (20 and 50 km) Marathon High-altitude sports Cycling Cross-country skiing Ice hockey Ice skating Biathlon
Swimming, water polo		

## The swimming issue: is swimming beneficial or detrimental for asthma?

Swimming has been considered for many years as a safe and healthy sport activity for children with asthma, due to the humid air inhaled during swimming thus reducing the risk of EIA, and suggested to have beneficial effects on disease severity (66). However, in recent years, several studies, especially from the group of Bernard, reported a potential risk of asthma with an increased swimming pool attendance in children (67). Other studies have demonstrated the association between the availability of chlorinated swimming pools and the prevalence of childhood asthma (68), independently of climate, altitude, and the socio-economic status of the country. The findings according to the ‘pool chlorine hypothesis’ postulate that the rise of childhood asthma may partly result from increased exposure of children to chlorine-based irritants, especially swimming pool disinfection by-products, such as trichloramine. Bernard et al. recently reported that asthma development during adolescents was clearly associated with cumulative pool attendance before the age 7 (69). This hypothesis is further supported by studies on occupational asthma in swimming pool workers and lifeguards (70, 71) and by studies comparing exposures to non-chlorinated pools (‘copper–silver pools’) (72) vs. chlorinated pools (73), in which attendance to the latter exerts a strong adjuvant effect to asthma and allergic rhinitis. A thorough evaluation of swimming and competing in chlorinated pools was recently published (74). Furthermore, very recent studies on mouse models of allergy showed hypochlorite-induced airway hyperreactivity, without evidence for allergic sensitization (75). All these studies are partly contradicted by a recent, large birth cohort study (the Avon Longitudinal Study of Parents and Children birth cohort) (76), in which British children from birth to the age of 10 did not increase their asthma risk with swimming pool attendance, and improved their lung function with a decreased risk of asthma symptoms.

However, although this question is unclear for development of asthma throughout childhood, competitive swimmers show an increase in asthma prevalence, with a mixed eosinophilic–neutrophilic airways inflammation (77, 78), epithelial damage (46), and very frequent bronchial hyperresponsiveness (79). Further, increased levels of leukotriene B4 have been reported in elite swimmers (77), supporting the hypothesis that repeated hyperventilation challenges (80) together with exposure to chlorine derivatives can contribute to a peculiar inflammation mechanism that may support the theory of a phenotype of its own for the ‘competitive swimmers’ asthma’, a syndrome that may be potentially reversible when the athlete quits the competitive activity (65, 81).

## Environmental issues

Various environmental factors may influence performance in different types of sports. Due to the almost daily repeated periods of high minute ventilation during the intense physical activity of training and competitions, typical for top athletes, they will have a higher exposure to possible pollutants and allergens in the environmental air. Different environmental factors may be important for different types of sports. For cold weather types of sports like cross-country skiing and biathlon, cold air may be the harmful environmental factor (15). For other types of sports, other environmental factors or pollutants may be important. For water sports taking place in swimming pools, organic chlorine products originating from chlorine used in the disinfection of the water in the pools are probably harmful (82), and, as already stated, bronchial hyperresponsiveness is frequently found in competitive swimmers (79) as well as airways inflammation (46, 77, 78). High levels of CO_2_, NO_2_, and ultrafine particles are often present in indoor ice rinks where propane- or gasoline-powered ice resurfacers and edgers are used (83, 84). Several studies demonstrated high prevalence of respiratory symptoms in ice-hockey players (85, 86), figure skaters (87), and speed skaters (88). Electric resurfacers, increased ventilation, and emission control systems have been recommended to reduce the exposure (89).

Outdoor sports may expose the athlete to environmental pollutants, and furthermore pollen and moulds may influence performances and the presence of EIB in allergic athletes (90). Pollution from traffic may influence air quality in athletic fields (91).

## Diagnosis: current guidelines

EIA should be suspected when cough, wheezing, and phlegm occur together with expiratory dyspnoea and audible rhonchi and sibilating rhonchi on lung auscultation after intense exercise of at least 5 min duration. Specific validated questionnaires can help to screen allergic athletes (92). Differential diagnoses must be considered (Table 2), bearing in mind that intense physical exercise may produce increased amounts of respiratory secretions that may mimic asthmatic symptoms (93). Thus, it is important to confirm the clinical suspicion by objective measurements in order to provide the optimal treatment for the athlete with respiratory problems.

**Table 2:** Exercise-induced asthma: differential diagnosis

Diagnosis	Relevant for:	Clinical presentation	Verification of diagnosis
EIA		Symptoms occur shortly after (sometimes during) physical exercise. The dyspnoea is of expiratory type. By auscultation: rhonchi and sibilating rhonchi. Respiratory retractions. Gradual improvement either spontaneously or after inhaled bronchodilator.	Exercise test with sub-maximal exercise load (95% load). Spirometry before and after exercise.
Exercise-induced laryngeal obstruction (EILO)	Asthmatics and individuals active in sports	Symptoms occur during maximum exertion. Symptoms disappear when exercise is stopped unless the patient continues to hyperventilate. The dyspnoea is of inspiratory type. There are audible inspiratory sounds from the laryngeal area and no signs of bronchial obstruction. No effect of pre-treatment with inhaled bronchodilator.	Exercise test with maximal exercise load, 6–8 min duration. Direct laryngoscopy during exercise test.
Exercise-induced hyperventilation	Individuals active in sports, general population	Hyperventilation with respiratory dyspnoea and decreased end-tidal CO_2_.	Case history, observation during dyspnoea.
Exercise-induced arterial hypoxemia (EIAH)	Individuals active in sports	Occurs in well-trained athletes with high maximum oxygen uptake. Thought to be due to diffusion limitations and ventilation–perfusion inequality. Incomplete diffusion in the healthy lung may be due to a rapid red blood cell transit time through the pulmonary capillaries.	Exercise test, sub-maximal to maximal level.
Swimming-induced pulmonary oedema (SIPE)	Individuals active in sports	May occur after heavy swimming exercises with symptoms of haemoptysis, cough, and respiratory distress. Reduced diffusion capacity (TLCO) for up to weeks afterwards.	Case history, clinical examination, and lung function measurements during an active episode.
Other chronic lung diseases	Individuals with chronic lung disease	Reduced baseline lung function may reduce physical performance due to limitations in airflow and lung volumes.	Exercise test with measurement of tidal flow volume loops during exercise.
Other general disease	Individuals with chronic illnesses – cardiovascular disorders	Chronic heart diseases and others general disorders.	General diagnostic workout.
Poor physical fitness including obesity	General population	Related to expectations. High heart rate after low-grade exercise load.	Exercise test: assessment of physical fitness by determination of *V’*O_2_max or maximal exercise load.

Modified with permission from (93).

Objective tests were necessary to obtain approval from the World Anti-Doping Association (WADA) or International Olympic Committee for the use of inhaled corticosteroids (ICSs) and inhaled β_2_-agonists in international competitive sports, but from 1st of January 2012 ICSs and the inhaled β_2_-agonists salbutamol, salmeterol and formoterol have been taken off the list of prohibited drugs and now do not have any restrictions for use in sports.

It has been claimed that a field exercise test is most likely to reproduce symptoms of the real-life exercise (94), but this has not been confirmed by other studies (45, 95). Furthermore, the field exercise test may be inconvenient both for logistical and standardization issues.

A standardized exercise test or other tests for bronchial hyperresponsiveness performed in a laboratory can be performed in a standardized way, including both environmental issues and exercise load by objective measures. Exercise tests can be performed by means of a motorized treadmill or a cycle ergometer, in a temperature- and humidity-controlled environment: the absolute water content should be below 10 mg H_2_O·L^−1^; otherwise, the test should be postponed until conditions become suitable (96). A very high workload is necessary to induce EIB which athletes can sometimes find difficult to reach in a lab environment. A key requirement is the ventilation reached and sustained with a target workload of 60–80% of the predicted Maximum Voluntary Ventilation that must be sustained for the last 4 min of an 8-min test (97). Heart rate recorded electronically or by electrocardiogram is also frequently used as a measure of exercise load. In a 6-min treadmill test, the speed of the treadmill is increased over the first 2 min to reach a level of 90–95% of maximum heart rate, which is sustained for the 4 min.

The objective tests of bronchial responsiveness are divided into ‘direct tests’ (methacholine, histamine) and ‘indirect tests’ (exercise, mannitol, adenosine 5′-monophosphate [AMP], non-isotonic aerosols, and hyperpnoea [EVH]). The methacholine (MCH) test is widely used. MCH acts as an analogue of acetylcholine, directly stimulating the cholinergic receptors in the airways’ smooth muscle. It has a high sensitivity but a low specificity for active asthma (45, 98), and a low sensitivity to identify EIB (99).

Mannitol is an osmotic agent that mimics the ‘osmolar’ mechanism of EIA/EIB, compares to the exercise test, and shows a similar sensitivity and specificity with MCH for the diagnosis of EIA/EIB (99–101).

The EVH test requires the subject to ventilate dry air containing ~5% CO_2_ for 6 min through a low-resistance circuit at a rate higher than that usually achieved on maximum exercise (102). Test is positive when a ≥10% sustained reduction in FEV1 is achieved. Very recent data, however, showed poor clinical reproducibility for the diagnosis of EIB in a cohort of recreational athletes (103), supporting the recommendations of a recent review in which bronchial provocation using both a direct and an indirect test is useful in some patients to confirm or exclude a diagnosis of asthma with certainty (104). A very basic exercise test that may help family doctors and paediatricians to suspect a primary diagnosis of EIA/EIB and refer the patient to a specialised centre is the ‘free-running asthma screening test’ (FRAST). FRAST measures peak expiratory flow rate before and at 1, 5, and 10 min after maximum voluntary running for at least 5 min, and it represents an acceptable, feasible, and cost-effective screening test (105), even if not in line with the most recent guidelines. In a recent study on elite swimmers it was suggested that parameters other than the direct drop in FEV compared to baseline should also be considered in the evaluation. Some subjects improve FEV during exercise with a subsequent drop after exercise providing a variability in FEV exceeding 15% (variability). Some subjects, previously without confirmed reversibility may respond to β_2_ with marked improved FEV compared to maximum drop after exercise (reversibility). Thus, when both direct EIB, significant variability or reversibility were considered, a closer association between symptoms and disease activity was found (106).

## Treatment: current guidelines

The treatment of EIA and of asthma in athletes should follow the same international guidelines as for the individual with general asthmatic symptoms. In all international guidelines for treating asthma in children and adults, a major aim of the treatment is to master EIA, as physical activity is seen as very important for the development and growth of children and for the self-perception. Fitness was found to correlate with psychological functioning in children with asthma (11). Considering that inflammation is the final result of the osmolar and vascular modifications described, anti-inflammatory treatment through inhaled steroids is often effective and sufficient to achieve a good EIA/EIB control (93). It should be noted that ICSs are the only anti-inflammatory drugs that improve respiratory epithelial healing (107). ICSs reduce the damage induced by repeated training and competitions, as we have seen for the phenotype of the ‘athlete's asthma’, enabling the athletes to master their sports and improving the long-term prognosis (10). However, a study in cross-country skiers showed no benefit from budesonide 800 µg/day during 3 months of treatment (108).

**Fig. 1:**
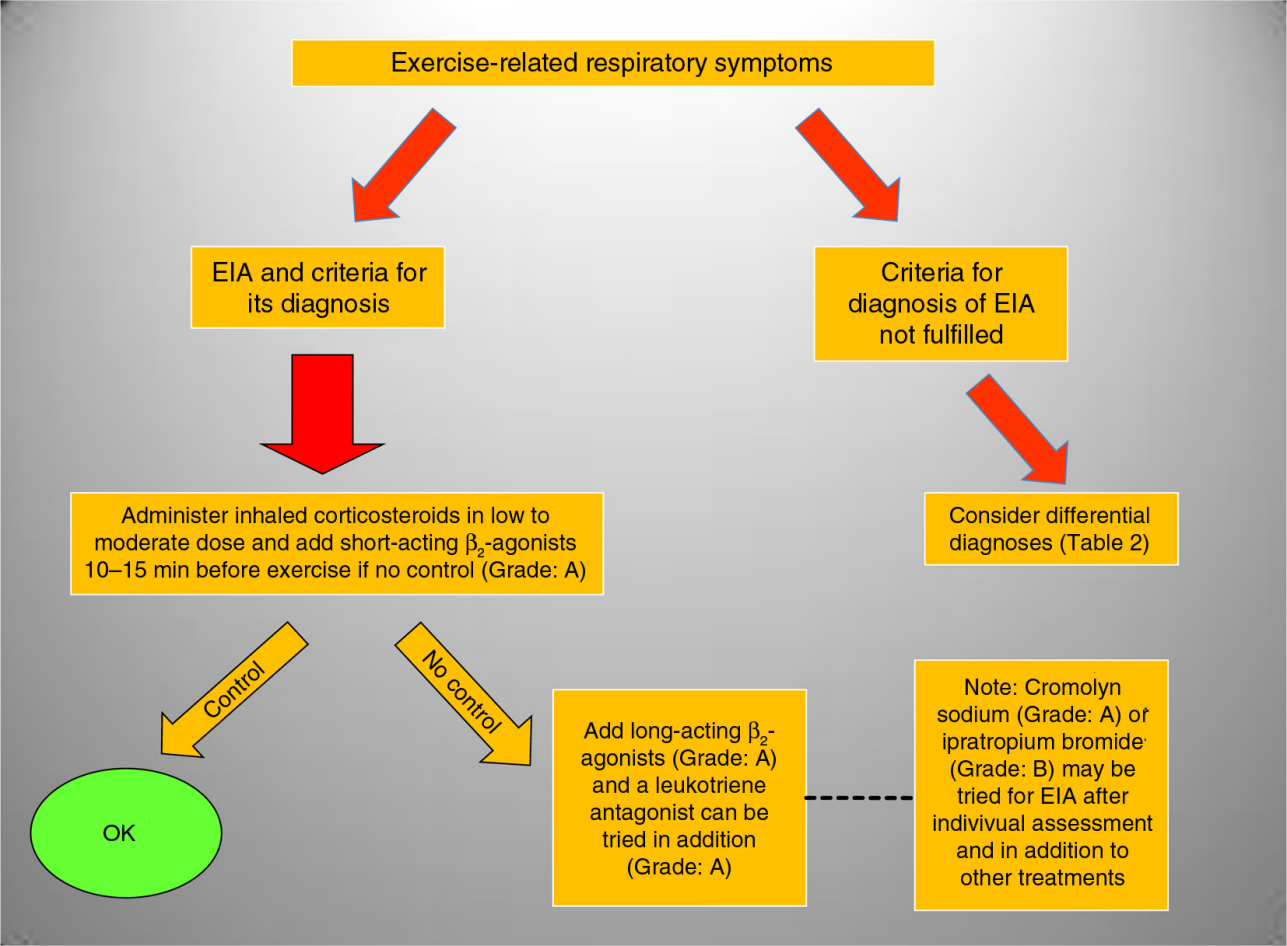
Simplified flow-chart for EIA treatment.

Inhaled short-acting β_2_-agonists are frequently needed and strongly suggested as pre-treatment before competition. If insufficient, long-acting β_2_-agonists (LABAs) and leukotriene antagonists may be added. Ipratropium bromide can be tried in addition to other treatments and after individual assessment (see flow chart in Fig. 1) (109). Based on the finding of increased parasympathetic tone in endurance athletes (110), the contribution of increased parasympathetic activity in the development of asthma in athletes would suggest a speculation for the role of inhaled ipratropium bromide or tiotropium in the treatment of asthma in athletes (15).

It is important to underline some concerns raised about tolerance of regular use of β_2_-agonists in EIA/EIB. First, there is a significant minority (15–20%) of asthmatics whose EIA is not prevented by β_2_-agonists, even when ICSs are used concomitantly; second, β_2_-agonists long-term regular use induces tolerance, with a decline in duration of the protective effect with their daily use, and lacks of sufficient safety data (111, 112). In addition, a recent report raised attention on a potential loss of bronchoprotection for athletes using LABAs, independent from the Arg16Gly polymorphisms that may affect the efficacy of these medications (113). Non-pharmacological measures are also of importance: nasal breathing and pre-exercise warm-ups (15–30 sec exertions alternate with 60–90 sec rest) followed by a warm-down segment are suggested (114), together with anti-cold masks for cold environments.

## Anti-doping: current regulations

For many years, the WADA issued strict regulations for the use of asthma drugs in sports. Initially, one feared that these drugs might improve performance, but after several studies on maximum performance in healthy subjects after inhaled β_2_-agonists, both short- and long-acting, it is generally accepted that inhaled steroids and inhaled β_2_-agonists do not improve performance. In a recent study combining three β_2_-agonists (salbutamol, formoterol, and salmeterol) all in WADA permitted doses, small but significant improvements could be seen in isometric quadriceps contraction and swim ergometric sprint performance. However, swim performance in an exhaustive race of 110 m did not improve (115). Since 1 January 2012, all ICSs have not been on the prohibited list, as well as the inhaled β_2_-agonists salbutamol, salmeterol, and formoterol. At present, there are no restrictions for the use of inhaled steroids; inhaled ipratropium bromide; leukotriene antagonists; and the inhaled β_2_-agonists salbutamol, salmeterol, and formoterol. Still, inhaled terbutaline is restricted in competitive sports, and objective measurements of AHR, EIB, or bronchodilator reversibility must be documented for approval of its use. Oral corticosteroids and oral or intravenous β_2_-agonists are prohibited. The list of prohibited drugs is usually updated every year and can be found on the WADA website (www.wada-ama.org).

## Exercise to improve exercise-induced symptoms: from animal models to field

**Fig. 2:**
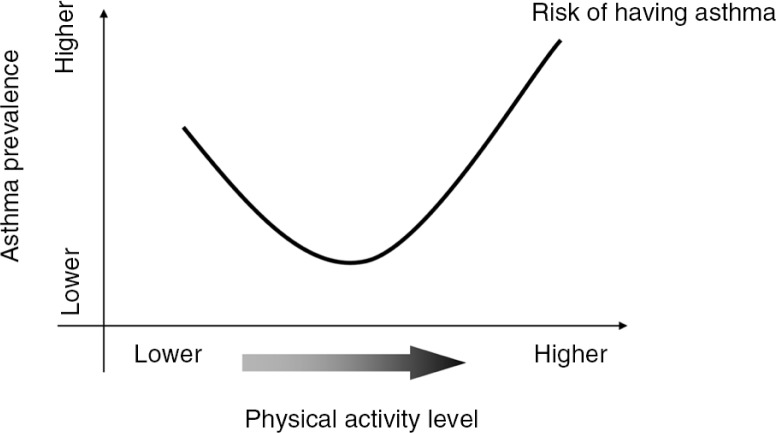
Suggested dose–response relationship between physical activity and asthma risk. (Courtesy of A. Moreira and L. Delgado, University of Porto, Portugal.)

At present, it is clear that strenuous exercise increases the risk for asthma development assuming a dose–response relationship between physical activity and EIA/EIB risk (see Fig. 2), with a ‘U’-shaped curve showing that moderate exercise training carries a lower risk of asthma in comparison to high-intensity exercise training especially endurance training and interval training. These observations are confirmed by a growing number of studies on the murine model of allergic asthma: low-to-moderate intensity aerobic exercise decreases eosinophilic and lymphocytic inflammation in mice exercising for 4 weeks, 5 days a week, at 50% exercise capacity (116). Aerobic exercise seems to reduce airway remodelling, with reduced airway smooth muscle hypertrophy and hyperplasia (116), a reduction in leukocyte infiltration, pro-inflammatory cytokine production, adhesion molecules expression (117), and enhanced regulatory T cell (Treg) responses (118). Aerobic exercise also shows an anti-inflammatory effect in mice exposed to air pollution (119). Furthermore, a single session of moderate aerobic exercise can decrease airway inflammation (but not bronchial responsiveness) in mice, with a down-regulation of inflammatory mediators’ genes expression and Th-2 derived cytokines production (120). Similar findings are also being demonstrated in humans, where a reduction in neutrophils count in patients with chronic inflammatory conditions has been observed (121). Another recent study on humans (122) showed that asthmatic patients subjected to aerobic exercise training have a reduction in the number of eosinophils in induced sputum and lower levels of FeNO. In addition, Moreira et al. also demonstrated, in children with persistent allergic asthma, that a physical training program did not increase airways inflammation but decreased their total and allergen-specific IgE levels (123). Finally, preliminary data show that regular exercise reduces IL-2 production, meaning that lymphocytes are probably less responsive to exogenous stimuli, and IL-4 producing lymphocytes are also reduced, suggesting a better clinical condition for allergic people that exercise regularly (124).

Therefore, it is apparent that aerobic, moderate-intensity exercise training (e.g. running or cycling) can be beneficial for allergic inflammation: these data open a new door on the possibility for ‘exercise therapy’ for asthmatics, in which exercise, in general a potential trigger for EIA/EIB, is instead a comprehensive part of the prevention and therapy strategies for asthmatics. However, on the other hand it has been shown that physical training programs in asthmatics improve cardiovascular fitness, but do not improve baseline lung function or bronchial hyperresponsiveness (125).

## Conclusion

EIA/EIB are highly common, and their prevalence is markedly increased in competitive athletes, especially within endurance sports. The role of swimming as an ‘asthmogenic’ or ‘non-asthmogenic’ sport in childhood is still debated, but for competitive swimmers sufficient evidence exists for the increased prevalence of asthma and bronchial hyperresponsiveness in young competitive swimmers. There are now convincing data implicating immune-mediated airway inflammation and epithelial damage in EIA/EIB pathogenesis together with an increased parasympathetic activity, and this improved understanding of the underlying mechanisms may lead to new treatments in terms of new drugs and different strategies focused on different therapeutic approaches based on different phenotypes and endotypes (126). Furthermore, murine models and preliminary studies on humans have demonstrated that exercise, despite being the cause of EIA/EIB, can also be a new tool for its treatment, and exercise prescriptions should be included in the treatment guidelines for EIA/EIB.
